# An Incidental Fallopian Tube Focal Serous Tubal Intraepithelial Lesion (STIL) Discovered on a Postoperative Pathology Report Following Hysterectomy and Salpingectomy: A Case Report

**DOI:** 10.7759/cureus.60992

**Published:** 2024-05-24

**Authors:** Arianne Shipp, Wanda I Torres

**Affiliations:** 1 Osteopathic Medicine, Nova Southeastern University Dr. Kiran C. Patel College of Osteopathic Medicine, Clearwater, USA; 2 Obstetrics and Gynecology, Nova Southeastern University Dr. Kiran C. Patel College of Osteopathic Medicine, Clearwater, USA; 3 Obstetrics and Gynecology, Suncoast Women's Care, Trinity, USA

**Keywords:** gynecological pathology report, gynecological laparoscopy, hysterectomy, salpingectomy, high-grade serous ovarian carcinoma, fallopian tube serous tubal intraepithelial lesion, stic, stil

## Abstract

A focal serous tubal intraepithelial lesion (STIL) is a rare lesion found on fallopian tubes that are characterized by atypical epithelial cells exhibiting morphological abnormalities with the accumulation of mutant p53 proteins. The p53 gene is a tumor suppressor gene, and when mutated gives rise to mutant p53 proteins that promote cancer cell growth and survival. We present a case of a 47-year-old gravida 2, para 2002 (G2P2) female who presented to the outpatient clinic with bilateral lower quadrant abdominal pain and back pain of four years' duration. The patient’s history included endometriosis with lysis of adhesions and gynecological laparoscopy, leiomyomata, infertility, ovarian cyst, dysmenorrhea, two full term births, and Essure implants used for contraception; her family history included maternal grandfather with breast cancer. Multiple fibroids and endometriosis were confirmed on pelvic ultrasound (US) and magnetic resonance imaging (MRI). Due to worsening pain, the patient chose to have an elective hysterectomy and Essure implant removal with bilateral salpingectomy. The postoperative pathology report revealed a right fallopian tube with a STIL. Multiple genetic mutations are known to contribute to the development of STILs including p53 and the breast cancer gene (BRCA). There are two BRCA genes, BRCA1 and BRCA2, that have many functions including producing proteins that repair damaged DNA. When mutated, this allows cells to divide and change rapidly, leading to certain types of cancer. Given the patient’s family history of breast cancer, the patient was tested for BRCA1 and BRCA2 for which the results were negative. However, even without having a BRCA mutation that is known to increase the risk of ovarian, fallopian tube, and peritoneal cancers, STILs continue to pose an increased risk of high-grade serous ovarian carcinoma (HGSOC). This case demonstrates the reasoning behind prophylactic salpingectomies alongside hysterectomies and the significance of the postoperative pathology report from gynecological procedures.

## Introduction

Female patients presenting with chronic lower quadrant abdominal pain and an enlarged uterus typically undergo pelvic ultrasound (US) to detect any structural abnormalities. If fibroids are identified on imaging, patients may receive a variety of treatments including but not limited to contraceptive methods, gonadotropin-releasing hormone agonists or antagonists, myomectomy, tranexamic acid, and uterine artery embolization. Those who refuse these less invasive treatments or continue to experience symptoms and do not improve following the treatments are given the option of a hysterectomy. As bilateral salpingo‐oophorectomy (BSO) reduces the risk of ovarian cancer by 80%-96%, bilateral salpingectomies have been increasingly suggested along with hysterectomies for benign gynecological conditions [1]. A BSO may be indicated in some cases, but is not always done, as removal of the ovaries results in immediate menopause [1]. However, it is becoming more commonly accepted that epithelial ovarian cancer most likely arises from the fallopian tube epithelium rather than from the ovary itself, giving bilateral salpingectomy with hysterectomy more merit [1].

Here, we present a case of a middle-aged female patient with chronic bilateral lower quadrant abdominal and pelvic pain who underwent a robot-assisted laparoscopic hysterectomy, bilateral salpingectomy, and right oophorectomy for fibroids, endometriosis, and Essure implant removal. The postoperative pathology report revealed crowding and overlapping of atypical epithelial cells lining the right fallopian tube, confirming the diagnosis of a focal serous tubal intraepithelial lesion (STIL). The patient followed up postoperatively with the gynecologic oncologist who recommended breast cancer gene (BRCA) testing as this could assist in determining the patient’s additional risk factors for high-grade serous ovarian carcinoma (HGSOC).

## Case presentation

A 47-year-old gravida 2, para 2002 (G2P2) female presented to the outpatient clinic in June 2023 with four years of chronic right lower quadrant abdominal pain, left lower quadrant abdominal pain, and back pain. The patient had a history of bilateral placement of the Essure implant for contraception. Her menstrual periods were described as monthly with five days of moderate flow accompanied by cramping and clotting. The patient’s gynecological exam revealed a fixed, tender, enlarged uterus with an irregular contour. There was no adnexal tenderness or mass palpated. The bilateral breast exam was normal with no tenderness, skin changes, nipple secretions, or masses palpated. A transabdominal and transvaginal pelvic US was ordered for an enlarged uterus. Pregnancy tests were negative at the time of the initial examination and prior to hysterectomy.

Her gynecological history included endometriosis with lysis of adhesions and gynecological laparoscopy, leiomyomata, infertility, ovarian cyst, dysmenorrhea, and two full term births. The age at menarche was 14, and the age of first sexual intercourse was 16. There was no history of sexually transmitted infections, diethylstilbestrol exposure, cervical dysplasia, vulvar dysplasia, human papillomavirus, abnormal colonoscopy, or abnormal mammogram. The patient reported undergoing dilation and curettage as a teenager. The patient’s medical history consisted of a hiatal hernia, chronic cholecystitis, vitamin D deficiency, iron deficiency anemia, hyperlipidemia, and hypothyroidism. Her family history included breast cancer in her maternal grandfather. Her social history was negative for tobacco use of any kind or any workplace/environmental exposures.

**Figure 1:**
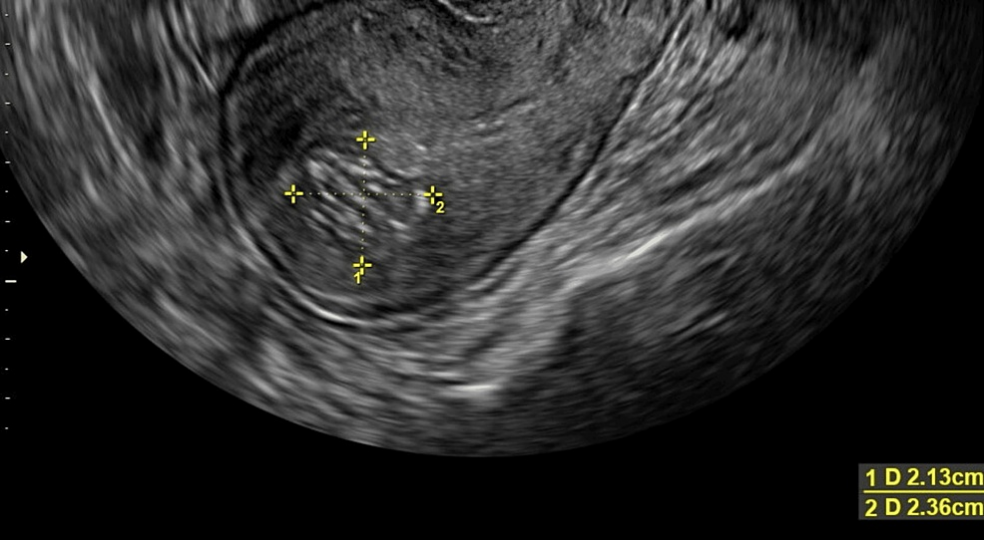
Ultrasound image showing a posterior intramural fibroid measuring 2.1 x 2.4 x 2.3 cm

**Figure 2:**
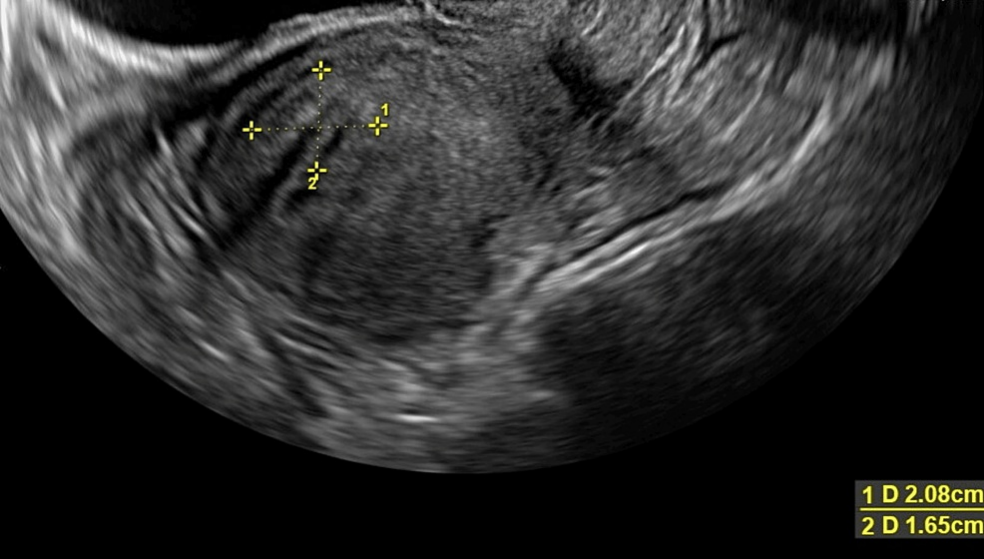
Ultrasound image showing an anterior fibroid measuring 2.1 x 1.7 x 1.9 cm

**Figure 3:**
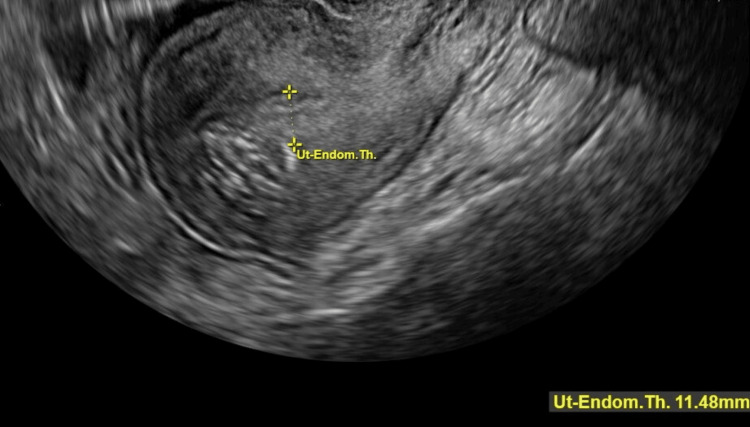
Ultrasound image showing the endometrial stripe measuring 11.48 mm

**Figure 4:**
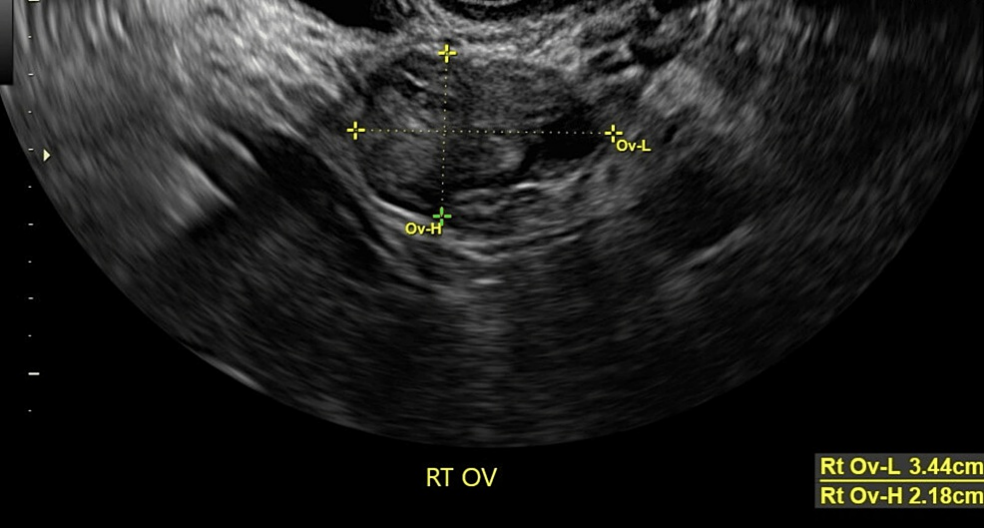
Ultrasound image showing the right ovary measuring 3.4 x 2.1 cm with a regular appearance

The pelvis, transabdominal, transvaginal US showed a posterior intramural fibroid measuring 2.1 x 2.4 x 2.3 cm and an anterior fibroid measuring 2.1 x 1.7 x 1.9 cm (Figures 1-2). The endometrial stripe measured 1.1 cm with a normal appearing endometrium and right ovary (Figures 3-4). The left ovary could not be visualized with certainty, and there was no free fluid throughout. A heterogeneous region in the right uterine cornua with shadowing was likely related to the prior Essure implant placement. After discussing these results with the patient, the patient requested removal of her Essure implants and surgical hysterectomy due to worsening pain. MRI of the pelvis with and without contrast was ordered prior to surgery. The results were reported as focal thickening of the uterine fundal junctional zone measuring 1.8 x 1.9 cm, concerning for either a focal adenomyoma or a submucosal fibroid. As per the MRI results, there was also a small endometriotic implant between the right ovary and an atypically positioned left ovary in the right adnexal region. Consent was obtained for a robotic-assisted total laparoscopic hysterectomy, bilateral salpingectomy, possible oophorectomy, cystoscopy, and cholecystectomy.

**Figure 5:**
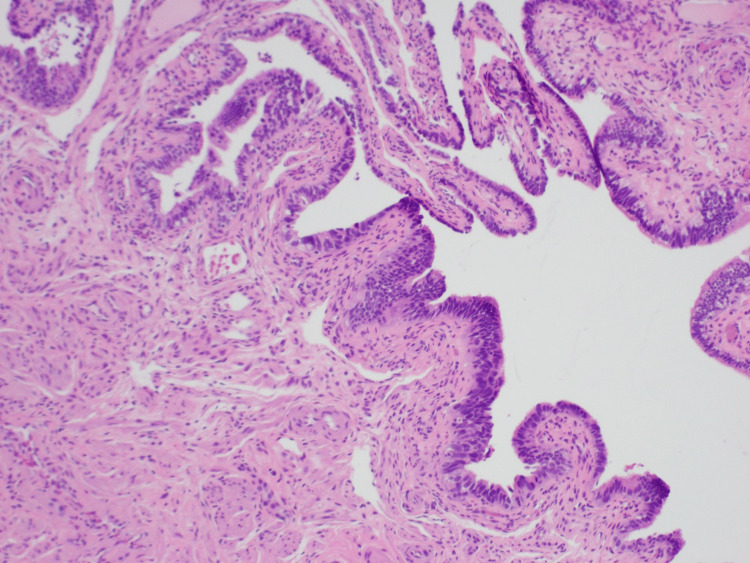
H&E staining of the STIL showing the crowding and overlapping of the atypical epithelial cells lining the fallopian tube compared to the normal epithelial cells in the background STIL, serous tubal intraepithelial lesion

**Figure 6:**
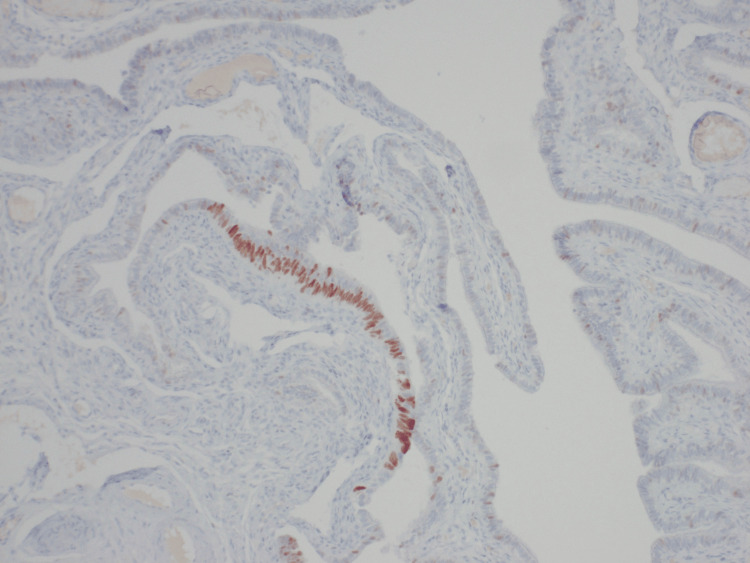
p53 immunostaining of the STIL diffusely highlighting the atypical cells of the fallopian tube confirming the diagnosis of the STIL, and only highlighting a few scattered normal epithelial cells in the background STIL, serous tubal intraepithelial lesion

During surgery, the patient received a total hysterectomy, bilateral salpingectomy, right oophorectomy and cholecystectomy due to chronic cholecystitis. The postoperative pathology report revealed the following: acute and chronic cervicitis with benign endocervical cysts; benign late secretory endometrium with adenomyosis; leiomyomata with the largest measuring 0.6 cm; right fallopian tube with a focal STIL and paratubal cyst; benign left fallopian tube and left ovarian remnant; benign right ovary with focal osseous metaplasia; and intact gallbladder with no masses or stones identified. Hematoxylin and eosin (H&E) staining showed the crowding and overlapping of the atypical epithelial cells lining the fallopian tube compared to the normal epithelial cells in the background (Figure 5). The p53 immunostain diffusely highlighted the atypical cells of the fallopian tube confirming the diagnosis of the STIL, and only highlighted a few scattered normal epithelial cells in the background (Figure 6). Metallic Essure devices were noted bilaterally in the right fallopian tube isthmus and the left cornu.

The patient had an unremarkable postoperative course. Given the identification of the focal serous tubal intraepithelial lesion, the patient was referred to a gynecologic oncologist. Per the gynecologic oncologist’s recommendation, the patient was tested for BRCA1 and BRCA2. The patient tested negative for both mutations.

## Discussion

Here, we have described a case of a 47-year-old female patient who initially presented with bilateral lower quadrant abdominal pain and back pain. After the US and MRI confirmation of fibroids and endometriosis, the patient underwent a total hysterectomy, bilateral salpingectomy, right oophorectomy, and excision of the left ovary remnant. The pathology results reported a STIL of the right fallopian tube.

STILs are characterized by an accumulation of mutant p53 proteins in more than 20 cells, some of which exhibit morphological abnormalities but normal to slightly increased proliferative activity [2]. When the p53 gene, a tumor suppressor gene, is mutated, this gives rise to mutant p53 proteins that promote cancer cell growth and survival. This patient’s pathology results reported confirmation of the STIL diagnosis given the H&E stain and p53 immunostain showing crowding and overlapping of the atypical epithelial cells lining the fallopian tube (Figures 1-2). A STIL differs from the serous tubal intraepithelial carcinoma (STIC) that involves a more aggressive dysregulation including many cells with architectural and nuclear alterations, upregulated proteins involved in cell adhesion and cancer invasion, mutant p53 proteins, and higher proliferative activity measured by the Ki-67 proliferative index [2]. Ki-67 is a protein found in dividing cells and can be used as a way to measure proliferation of cancer cells. Ki-67 is found in greater than 10% of cells in STICs, and varies in STILs [2].

High-grade serous ovarian carcinoma has been associated with STIC/STIL with multiple lesions in the fimbria, older age, and many p53 signatures (benign-appearing tubal epithelium with a p53 mutation) [3]. The current theory of HGSOC pathogenesis starts with fallopian tube secretory cell outgrowth (SCOUT) and progresses through the p53 signature to STILs and STICs [4]. It is believed that STIC cells detach from the fallopian tube surface and disseminate to the ovaries and peritoneal tissue, where masses are formed [3]. STICs have been identified in the fallopian tube of up to 59% of female patients with HGSOC as well as in primary peritoneal serous carcinomas [3]. STILs are very similar to STICs, but fall short of being a direct precursor to HGSOC due to their limited proliferation. However, it has been hypothesized that STILs represent exfoliated precursor cells that may eventually undergo malignant transformation leading to ovarian cancer [5]. Examination and sectioning of the fallopian tube is done to detect any cancerous or precancerous lesions such as STICs and STILs and may decrease the overall risk of HGSOC [6]. This is also the reason mentioned in the current literature for performing prophylactic salpingectomies alongside hysterectomy for benign indications [7].

Additionally, ovarian cancers have been associated with many gene mutations and aberrant protein expression including but not limited to p53, p21, cyclin E1, Rsf-1, laminin γ1 protein, fatty acid synthase, stathmin1, p16, and BRCA [3]. BRCA1 and BRCA2 have many functions including producing proteins that repair damaged DNA and regulate the cell cycle. When mutated, this allows cells to divide and change rapidly, leading to cancer progression. The supporting evidence states carriers of the BRCA1 and BRCA2 mutations contribute to the survivability of lesional cells by increased triglycerides, preserving homeostasis, and facilitating the development of STICs and STILs [5]. Female patients with BRCA1 or BRCA2 mutations are at an increased risk of ovarian, fallopian tube, and peritoneal cancers [8]. As this patient had a known family history of breast cancer, it was imperative to follow up with BRCA testing that came out negative.

## Conclusions

This case demonstrates the importance of pathology staining in gynecological surgical cases such as this one. The discovery of the STIL along with the bilateral salpingectomy and right oophorectomy could have prevented the development of an invasive cancer in this patient. We propose that the fimbrial end of the fallopian tube should continue to be routinely sectioned and examined as it provides the opportunity to detect any early malignant changes and decrease the overall risk of HGSOC. This case is an example of why there are increasing prophylactic salpingectomies alongside hysterectomy for benign indications. The presentation of this patient also shows the lack of specific symptomatology in a developing internal precancerous lesion, again highlighting the importance of preventative removal and staining of the fallopian tubes. Additionally, the documentation of this patient’s personal and family history along with the STIL findings on the pathology report contributes to the growing knowledge of STIL cases as current information regarding such lesions is limited.
